# Association between coronary plaque volume and myocardial ischemia detected by dynamic perfusion CT imaging

**DOI:** 10.3389/fcvm.2022.974805

**Published:** 2022-09-08

**Authors:** Borbála Vattay, Sarolta Borzsák, Melinda Boussoussou, Milán Vecsey-Nagy, Ádám L. Jermendy, Ferenc I. Suhai, Pál Maurovich-Horvat, Béla Merkely, Márton Kolossváry, Bálint Szilveszter

**Affiliations:** ^1^Cardiovascular Imaging Research Group, Heart and Vascular Center, Semmelweis University, Budapest, Hungary; ^2^Medical Imaging Center, Semmelweis University, Budapest, Hungary

**Keywords:** dynamic perfusion CT, myocardial blood flow, coronary computed tomography, coronary plaque volume, quantitative plaque analysis

## Abstract

**Introduction:**

We aimed to evaluate the relationship between quantitative plaque metrics derived from coronary CT angiography (CTA) and segmental myocardial ischemia using dynamic perfusion CT (DPCT).

**Methods:**

In a prospective single-center study, patients with > 30% stenosis on rest CTA underwent regadenoson stress DPCT. 480 myocardium segments of 30 patients were analyzed. Quantitative plaque assessment included total plaque volume (PV), area stenosis, and remodeling index (RI). High-risk plaque (HRP) was defined as low-attenuation plaque burden > 4% or RI > 1.1. Absolute myocardial blood flow (MBF) and relative MBF (MBFi: MBF/75th percentile of all MBF values) were quantified. Linear and logistic mixed models correcting for intra-patient clustering and clinical factors were used to evaluate the association between total PV, area stenosis, HRP and MBF or myocardial ischemia (MBF < 101 ml/100 g/min).

**Results:**

Median MBF and MBFi were 111 ml/100 g/min and 0.94, respectively. The number of ischemic segments were 164/480 (34.2%). Total PV of all feeding vessels of a given myocardial territory differed significantly between ischemic and non-ischemic myocardial segments (*p* = 0.001). Area stenosis and HRP features were not linked to MBF or MBFi (all *p* > 0.05). Increase in PV led to reduced MBF and MBFi after adjusting for risk factors including hypertension, diabetes, and statin use (per 10 mm^3^; β = −0.035, *p* < 0.01 for MBF; β = −0.0002, *p* < 0.01 for MBFi). Similarly, using multivariate logistic regression total PV was associated with ischemia (OR = 1.01, *p* = 0.033; per 10 mm^3^) after adjustments for clinical risk factors, area stenosis and HRP.

**Conclusion:**

Total PV was independently associated with myocardial ischemia based on MBF, while area stenosis and HRP were not.

## Introduction

Currently luminal stenosis is the most dominant factor in the management of coronary artery disease (CAD) (1). Quantitative plaque assessment and adverse plaque characteristics may further improve cardiovascular risk prediction and patient management (2). Furthermore, anatomical and functional assessment of CAD could also improve clinical outcomes (3), however, the link between stenosis severity and myocardial ischemia is controversial (4).

CT angiography (CTA) is a uniquely suited imaging modality that can simultaneously evaluate plaque morphology and ischemia (5). Also, CTA allows accurate characterization and quantification of coronary plaques over stenosis assessment. Moreover, myocardial dynamic perfusion CT (DPCT) provides functional data and can quantitatively assess myocardial perfusion during pharmacological stress (6).

Former observational studies evaluated the link between coronary plaque burden and global myocardial ischemia using qualitative/visual assessment by either static CT perfusion (CTP) (7), stress echocardiography (8) or SPECT (9). Based on these studies, whether stenosis severity, adverse plaque features or coronary plaque burden is predictive for ischemia remains uncertain. Also, it is unknown whether quantitative plaque characterization can predict segmental ischemia as assessed by quantitative DPCT imaging. Previous studies exclusively reported vessel-based data, however, we applied a novel segment-based analysis considering only coronary lesions corresponding to myocardial territories.

Our aim was to elucidate the association between quantitative atherosclerotic plaque metrics derived from coronary CTA and segmental myocardial ischemia based on myocardial blood flow (MBF) as detected by DPCT imaging.

## Materials and methods

### Study population and protocol

**FIGURE 1:**
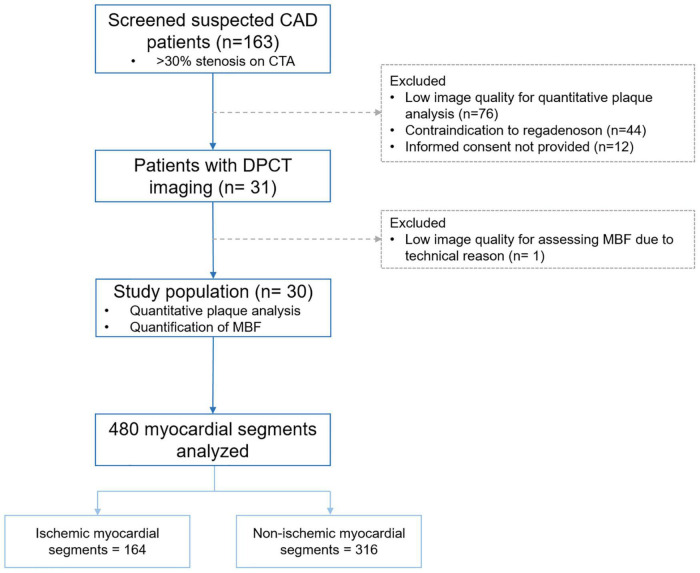
Flow-chart of the study. CAD, Coronary artery disease; CTA, CT angiography; DPCT, Dynamic perfusion CT; MBF, Myocardial blood flow.

Patients with stable chest pain and > 30% coronary stenosis detected on rest CTA were screened for our prospective, single-center study. Inclusion criteria were at least 30% stenosis in one of the main coronary arteries and excellent image quality for the quantitative analysis of the whole coronary tree. Exclusion criteria were prior myocardial infarction or revascularization, heart transplantation, contraindication to regadenoson or low image quality for quantitative assessment of coronary lesions. Regadenoson stress DPCT was performed at a separate appointment after written informed consent was obtained from all patients. Subjects with low image quality for the assessment of myocardial ischemia were excluded (*n* = 1). Patients were enrolled in the analysis if found eligible based on inclusion and exclusion criteria. Flow chart of the study is shown in Figure 1.

The study was approved by the national ethical committee (National Institute of Pharmacy and Nutrition—OGYÉI/719/2017) and was performed in accordance with the Helsinki declaration.

Demographic data and comorbidities were collected by reviewing patients’ medical records. Hypertension was determined as systolic blood pressure > 140 mmHg and/or diastolic blood pressure > 90 mmHg based on office measurements or the use of antihypertensive therapy. Diagnosis of hyperlipidemia was based on total cholesterol level > 200 mg/dL or the administration of lipid-lowering medication. Diabetes mellitus was defined as elevated plasma glucose levels (fasting plasma glucose ≥ 126 mg/dL; HbA1C ≥ 6.5%) or the use of antidiabetic medication or insulin therapy.

### Coronary CT angiography protocol

Prospectively triggered CTA scan of the heart was performed according to the guidelines of the Society of Cardiovascular Computed Tomography (SCCT) with a 256-slice multidetector row CT (Brilliance iCT, Philips Healthcare, Cleveland, OH, United States) (10). *Per os* beta blocker was administered 1 h prior examination if the heart rate (HR) was above 65 beats/min. All patients received 0.8 mg of sublingual nitroglycerine before CTA scanning if systolic blood pressure was > 100 mmHg, and in case of HR > 60 beats per minute intravenous beta blocker was additionally administered. Image acquisition was performed at diastole (75–81% of the R–R interval) or at systole (37–43% of the R–R interval) in case of HR > 70 beats per minute despite premedication. The following scan parameters were applied: 270 ms gantry rotation time, 128 × 0.625 mm collimation, tube voltage 100–120 kVp, and tube current 200–300 mAs based on patient’s body mass index (BMI). A four-phasic contrast injection protocol was used with 85–95 ml contrast agent at a flow rate of 4.5–5.5 ml/s. Axial images were reconstructed with 0.6 mm slice thickness using iterative reconstruction (iDose4 Level 5, Philips Healthcare, Cleveland, OH, United States).

### Dynamic perfusion CT protocol

Stress DPCT scan was performed after rest CTA at a separate appointment with the same scanner. Hyperemia was induced using single dose of 400 μg intravenous regadenoson (Rapiscan^®^, GE Healthcare) (11). Stress acquisition was performed during a single breath-hold in inspiration, 1 min after bolus regadenoson was administered during peak stress covering 25–30 cardiac cycles (12). Patients’ HR, oxygen saturation and blood pressure were monitored to confirm appropriate levels of stress for CTP imaging. Contrast injection protocol included 50–60 ml contrast bolus at an infusion rate of 5 ml/s, followed by 30 ml saline chaser. Prospective electrocardiogram (ECG)-gated dynamic mode (with 64 × 1.25 mm collimation, 360° reconstruction, 8 cm coverage) was acquired in systolic phase (35% of the RR interval), with tube voltage of 80–120 kVp and tube current of 100–250 mAs based on patient’s BMI. Images were reconstructed using hybrid iterative reconstruction (iDOSE4 level 5, Philips Healthcare, Cleveland, OH, United States) with 2.0 mm slice thickness and 2.0 mm increment.

### Quantitative plaque analysis

Coronary artery segments were defined using an 18-segment model as recommended by the SCCT guidelines (10). CTA images were transferred into a dedicated software tool (QAngioCT Research Edition v3.1; Medis Medical Imaging Systems, Leiden, The Netherlands) for quantitative plaque analysis. Images were analyzed by a single reader (BV, 3 years of experience with cardiac CT) blinded to patient’s data and perfusion parameters. The software automatically extracted the coronary tree. All coronary vessels with a diameter > 1.5 mm were evaluated. After automatic contouring of the lumen and vessel wall, manual correction was performed—if needed—in both longitudinal and cross-sectional views at 0.5 mm increments. The proximal and distal borders of coronary plaques were defined for quantification. Coronary plaque was defined on the CTA based on former publications (13). Chronic total occlusions were not present in current patient population. Plaque composition was determined using fixed thresholds: low-attenuation plaque (LAP): −100–30 HU; non-calcified plaque (NCP): 31–350 HU; calcified plaque (CP): ≥ 351 HU. Volumes of total plaque, LAP, NCP and CP were calculated. LAP burden defined as the ratio of LAP volume and vessel volume (LAP volume × 100%/vessel volume) was also determined. Lumen area stenosis was defined at the site of the maximal luminal stenosis of the coronary plaque. Remodeling index (RI) was calculated as the ratio of the vessel wall area at the site of the maximal luminal narrowing and the reference vessel wall area. High-risk plaque (HRP) was defined based on quantitative LAP burden > 4% or a RI > 1.1 (2, 14).

### Myocardial perfusion analysis

DPCT images were analyzed using a dedicated software (Intellispace Portal; Philips Healthcare, Cleveland, OH, United States). Elastic registration and temporal filtering were applied for motion artifact reduction. Time-attenuation curves (TAC) created in the left ventricular outflow tract were used as arterial input function for perfusion analysis. Short-axis views were created for the assessment of the left ventricular myocardial tissue. MBF was computed applying a hybrid deconvolution method (12). The assessment of MBF was obtained by two readers (B.V and S.B, 3 and 4 years of experience with cardiac CT) in random order blinded to plaque data and patient characteristics. A ROI > 0.5 cm2 was set in each myocardial segment (intramural) using a 16-segment model excluding the apex carefully avoiding any artifacts on short-axial images (15). Segmental myocardial ischemia was defined as MBF < 101 ml/100 g/min based on Pontone et al. (16). In addition, relative MBF (MBFi) for each segment was also calculated as the ratio of absolute MBF to reference MBF, latter defined as the 75th percentile of all MBF values of a given patient (17).

### Integration of coronary anatomy and myocardial territories

**FIGURE 2:**
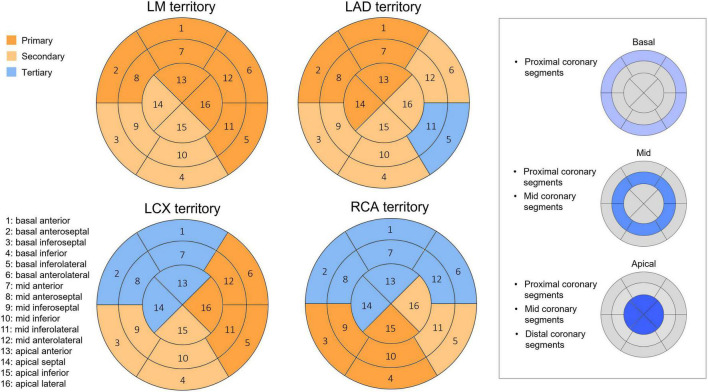
Integration of coronary anatomy and myocardial territories. Vessel territories were defined based on the modified method after Cerci et al. for LM, LAD, LCX, and RCA. The following categories were determined for myocardial segments: primary—most commonly supplied territories in case of right dominance; secondary—might be supplied territories in case of normal variations; tertiary—usually not supplied territories. In addition, coronary segment-based analysis was also used, taking lesion location into account. For that, basal segments were aligned with proximal, mid segments with proximal and mid, and apical segments with proximal, mid and distal coronary segments. LAD, Left anterior descending; LCX, Left circumflex; LM, Left main; RCA, Right coronary artery.

Coronary lesions were assigned to the corresponding myocardial segment based on the modified method after Cerci et al. for the CORE320 (Coronary Artery Evaluation Using 320-Row Multidetector CTA) trial (18). Former studies performed vessel-based analysis for the alignment of myocardial territories and supplying vessels. For our segment-based approach, we defined all coronary artery segments that supply a given myocardial segment of the 16 analyzed segments based on dominance, segment location in relation to basal, mid-ventricular or apical regions (Figure 2).

After the adjudication was performed by B.S.,—with 8 years of experience in cardiac imaging—volumes for total, NCP and CP of all relevant supplying coronary segments were summed for each myocardial segment. LAP burden was also calculated from the summed LAP and vessel volume. If LAP burden exceeded 4% of all plaque supplying a given segment, or the highest RI of the corresponding lesions was > 1.1, we marked as HRP. Summed plaque volumes (PVs), the highest degree of lumen area stenosis and HRP (LAP burden > 4% or a RI > 1.1) of the supplying coronary segments were analyzed for the corresponding myocardial segment.

### Statistical analysis

Continuous variables are presented as mean and standard deviation, whereas categorical parameters are presented as frequency with percentages. Independent *t*-test was used to compare parameters describing coronary plaque burden between ischemic and non-ischemic segments. Pearson correlation was used to define the association between total, NCP and CP volumes. Linear and logistic mixed models correcting for intra-patient clustering and clinical factors were used to assess the association between total PV, maximal area stenosis, quantitative HRP features and absolute MBF, MBFi or myocardial ischemia using 101 ml/100 g/min as cut-off value for MBF. Models were adjusted for predefined clinical risk factors of CAD and possible modifiers of ischemia including hypertension, diabetes mellitus and statin therapy.

Intraclass correlation coefficient (ICC) of MBF was calculated for 160 segments of 10 randomly selected patients between two readers with 3 or more years of experience in cardiac CT imaging (BV and SB). ICC values greater than 0.80 were considered good, values above 0.90 were considered to have excellent reproducibility. Also, reproducibility of quantitative plaque assessment was evaluated between two independent readers based on 10 plaques of randomly selected patients. All statistical analyses were performed using SPSS (version 24.0) and R software (version 3.6.1). *P* < 0.05 was defined as statistically significant.

## Results

### Patient characteristics

The baseline characteristics of the 30 analyzed patients (mean age 60.9 ± 8.3 years, 26.7% female, mean BMI 28.9 ± 3.8 kg/m^2^) are summarized in Table 1. Common comorbidities were hypertension (76.7%) and dyslipidemia (76.7%).

**TABLE 1:** Patient characteristics.

	Patient population *N* = 30
Age, years	60.9 ± 8.3
Male gender, *n* (%)	22 (73.3)
BMI, kg/m^2^	28.9 ± 3.8
Hypertension, *n* (%)	23 (76.7)
Diabetes mellitus, *n* (%)	2 (6.7)
Dyslipidemia, *n* (%)	23 (76.7)
Smoking, *n* (%)	16 (53.3)
Cerebrovascular disease, *n* (%)	1 (3.3)
Peripheral artery disease, *n* (%)	3 (10.0)
Family history of premature CAD, *n* (%)	9 (30.0)
Oral anticoagulant therapy, *n* (%)	6 (20.0)
Statin therapy, *n* (%)	16 (53.3)
ACE-I/ARB therapy, *n* (%)	18 (60.0)
Beta-blocker therapy, *n* (%)	15 (50.0)

Continuous variables are described as mean ± SD, whereas categorical variables are represented as frequencies and percentage.

ACE-I, Angiotensin-converting-enzyme inhibitor; ARB, Angiotensin receptor blocker; BMI, Body mass index; CAD, Coronary artery disease.

On average, 13.0 ± 8.6 days have passed between the two examinations. Mean effective radiation dose was 4.4 ± 1.1 mSv for rest CTA and 8.9 ± 4.0 mSv for DPCT. A total of 496 coronary artery segments and 480 myocardial segments were evaluated quantitatively. ICC between readers was 0.96 and 0.93 for MBF and total PV, respectively.

### Plaque characteristics and segmental myocardial ischemia

**FIGURE 3:**
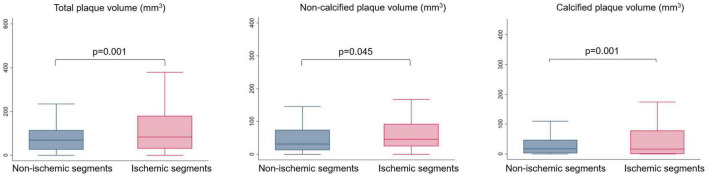
Box plots showing the distribution of total, non-calcified and calcified plaque volumes related to ischemic and non-ischemic myocardial segments based on DPCT (MBF < 101 ml/100 g/min vs. ≥ 101 ml/100 g/min). DPCT, Dynamic perfusion CT; MBF, Myocardial blood flow.

Total PV, NCP volume, and CP volume differed significantly between ischemic and non-ischemic myocardial segments, 120.5 ± 119.5 mm^3^ vs. 84.6 ± 82.2 mm^3^, *p* = 0.001; 62.3 ± 59.5 mm^3^ vs. 51.4 ± 54.9 mm^3^, *p* = 0.045; 58.3 ± 91.8 mm^3^ vs. 33.3 ± 50.6 mm^3^, *p* = 0.001; respectively (Table 2). Median and interquartile range (IQR) of PVs for ischemic and non-ischemic myocardial segments were: total PV: 82.9 (31.1–179.6) vs. 68.7 (25.8–114.7) mm^3^; NCP volume: 46.1 (24.3–93.7) vs. 31.6 (12.4–73.8) mm^3^; CP volume: 15.9 (0.1–78.2) vs. 17.3 (2.2–46.1) mm^3^. Figure 3 demonstrates box plots of quantitative PVs in coronary segments supplying ischemic and non-ischemic myocardial segments. On a patient level, the average of maximal lumen area stenosis of the worst lesion was 54.7 ± 15.9%. On a segmental level, the average of the maximal lumen area stenosis was 37.2 ± 22.7% for ischemic and 33.5 ± 20.7% for non-ischemic myocardial segments (*p* = 0.072). HRP was present in 21.3% in ischemic and 19.0% in non-ischemic territories (*p* = 0.539).

**TABLE 2:** Coronary plaque characteristics in ischemic and non-ischemic myocardial segments.

	Ischemic myocardial segments *N* = 164	Non-ischemic myocardial segments *N* = 316	*P*
Total plaque volume, mm^3^	120.5 ± 119.5	84.6 ± 82.2	**0.001**
NCP volume, mm^3^	62.3 ± 59.5	51.4 ± 54.9	**0.045**
CP volume, mm^3^	58.3 ± 91.8	33.3 ± 50.6	**0.001**
High-risk plaque, *n* (%)	35 (21.3)	60 (19.0)	0.539
Lumen area stenosis,%	37.2 ± 22.7	33.5 ± 20.7	0.072

Myocardial ischemia was defined as MBF < 101 ml/100 g/min. Continuous variables are described as mean ± SD, whereas categorical variables are represented as frequencies and percentage.

Bold values indicate significant differences based on the p-values.

CP, Calcified plaque; MBF, Myocardial blood flow; NCP, Non-calcified plaque.

Number of ischemic segments were 164/480 (34.2%). Median MBF was 111 ml/100 g/min, while median MBFi was 0.94.

Total PV strongly correlated with NCP volume (*r* = 0.73, *p* < 0.001) and CP volume (*r* = 0.83, *p* < 0.001), we therefore included total PV in the multivariate prediction models to avoid multicollinearity.

### Predictors of absolute and relative myocardial blood flow

Using linear mixed models, univariate analysis revealed that total PV predicted both absolute and relative MBF values (Table 3). Clinical risk factors (including hypertension, diabetes mellitus, and statin use), HRP and stenosis severity were not associated with impaired myocardial perfusion based on MBF and MBFi.

**TABLE 3:** Univariate analysis of the predictors of absolute and relative myocardial blood flow (MBFi) detected by DPCT using linear mixed models.

Predictors	Absolute MBF detected by DPCT			Relative MBF detected by DPCT		
		
	Univariate model			Univariate model		
		
	β	95% CI	*P*	β	95% CI	*P*
Total plaque volume, per 10 mm^3^	**-0.025**	**-0.043—0.007**	**0.006**	**-0.0002**	**-0.0003—0.0001**	**0.004**
NCP volume, per 10 mm^3^	−0.025	−0.053–0.003	0.079	−0.0002	−0.0004–0.0000	0.077
CP volume, per 10 mm^3^	**-0.046**	**-0.078—0.014**	**0.005**	**-0.0002**	**-0.0004—0.00006**	**0.008**
Remodeling index	1.934	−2.427–6.295	0.384	0.003	−0.029–0.036	0.838
High-risk plaque	1.952	−1.767–5.672	0.303	0.018	−0.009–0.045	0.191
Lumen area stenosis	−4.479	−12.008–3.050	0.243	−0.042	−0.093–0.009	0.108
Age, years	−0.172	−1.208–0.864	0.736	0.0006	−0.001–0.002	0.463
BMI, kg/m^2^	−0.791	−3.037–1.455	0.477	**0.004**	**0.0007–0.007**	**0.017**
Hypertension	−1.482	−21.491–18.527	0.881	0.010	−0.019–0.039	0.491
Diabetes mellitus	1.542	−32.393–35.478	0.926	0.018	−0.031–0.068	0.456
Smoking	1.536	−15.424–18.496	0.854	0.013	−0.011–0.038	0.274
Statin therapy	−3.201	−20.127–13.724	0.701	−0.005	−0.030–0.020	0.658

Bold values indicate significant differences based on the p-values.

CP, Calcified plaque; BMI, Body mass index; DPCT, Dynamic perfusion CT; MBF, Myocardial blood flow; MBFi, Myocardial blood flow index; NCP, Non-calcified plaque.

On multivariate analysis, total PV increase led to reduced absolute and relative MBF values even after adjusting for clinical risk factors, lumen area stenosis and HRP features: per 10 mm^3^; β = −0.035, *p* < 0.01 for MBF and β = −0.0002, *p* < 0.01 for MBFi. Notably, lumen area stenosis and quantitative HRP features were not linked to absolute or relative MBF values (all *p* > 0.05).

### Predictors of myocardial ischemia based on myocardial blood flow threshold

On univariate logistic regression total PV and lumen area stenosis were significant predictors of myocardial ischemia based on MBF < 101 ml/100 g/min (Table 4). After adjusting for predefined clinical risk factors, stenosis severity and HRP, increase in total PV was independently associated with myocardial ischemia: OR: 1.01, *p* = 0.033 (per 10 mm^3^). However, on multivariate analysis HRP feature and lumen area stenosis were not linked to ischemia (both *p* > 0.05).

**TABLE 4:** Univariate logistic regression analysis of the predictors of myocardial ischemia detected by DPCT.

	Myocardial ischemia		
Predictors	detected by DPCT		
	
	Univariate model		
	
	OR	95% CI	*P*
Total plaque volume, per 10 mm^3^	**1.01**	**1.002–1.012**	**0.003**
NCP volume, per 10 mm^3^	**1.01**	**1.004–1.018**	**0.002**
CP volume, per 10 mm^3^	1.01	0.997–1.015	0.172
Remodeling index	1.14	0.422–3.059	0.801
High-risk plaque	0.79	0.333–1.890	0.601
Lumen area stenosis	**8.05**	**1.340–48.333**	**0.023**
Age, years	1.03	0.839–1.268	0.770
BMI, kg/m^2^	1.26	0.787–2.019	0.335
Hypertension	2.86	0.046–177.116	0.617
Diabetes mellitus	1.74	0.003–883.279	0.861
Smoking	0.96	0.031–29.434	0.982
Statin therapy	4.06	0.108–152.174	0.448

Myocardial ischemia was defined as MBF < 101 ml/100 g/min.

Bold values indicate significant differences based on the p-values.

CP, Calcified plaque; BMI, Body mass index; DPCT, Dynamic perfusion CT; MBF, Myocardial blood flow; NCP, Non-calcified plaque.

## Discussion

We used a novel approach to define the contribution of coronary PV to limited flow (ischemia) of all feeding coronary segments considering only coronary lesions prior to a given myocardial territory. We established that total PV influenced myocardial perfusion on a segmental level, independent from stenosis severity, HRP and risk factors. Moreover, maximal luminal area stenosis and the presence of HRP were not linked to myocardial ischemia based on MBF. Reproducibility was excellent for the evaluation of MBF or total PV.

While there are several alternative imaging modalities to analyze the hemodynamic consequence of coronary plaques, CT is the only non-invasive modality for the combined assessment of morphology and function of CAD. CT can provide several additional anatomical parameters that could be incremental as compared with traditional evaluation focusing on luminal stenosis or lesion length. In agreement with our findings, total PV was linked to visual perfusion defects as assessed by SPECT (9, 19). Liu et al. also reported that low-density PV and diameter stenosis were also independently associated with myocardial ischemia. Driessen et al. evaluated 208 patients who underwent (15O) H_2_O PET-MPI and coronary CTA and found that plaque length and volume were inversely associated with MBF in a sub-study of the PACIFIC trial (20). Moreover, this study suggested a link between decreased flow and NCP volume or positive remodeling in a vessel-based analysis. The multicenter CORE 320 study demonstrated that combined CTA and CTP has excellent diagnostic performance to detect flow-limiting lesions (more than 50%) by invasive angiography and perfusion defects by SPECT. van Rosendael et al. utilized static stress CTP in a total of 84 patients to evaluate the relationship between morphological plaque features and visual perfusion deficits (7). Interestingly, increasing stenosis severity and lesion length were predictors of ischemia, however PVs were not. Previous studies examining the association between PV and ischemia in stable angina patients reported highly variable mean values for total PV: 69.0 ± 16.8 mm^3^ vs. 49.6 ± 17.2 mm^3^ by van Rosendael et al. (7), 114 ± 118 mm^3^ vs. 62 ± 89 mm^3^ by Diaz-Zamudio et al. (9), and 694.6 ± 485.1 mm^3^ vs. 422.3 ± 387.9 mm^3^ by Min et al. (21), for ischemic and non-ischemic myocardial territories, respectively. In our current study, total PV for ischemic segments were 120.5 ± 119.5 mm^3^ while for non-ischemic segments it was 84.6 ± 82.2 mm^3^.

**FIGURE 4:**
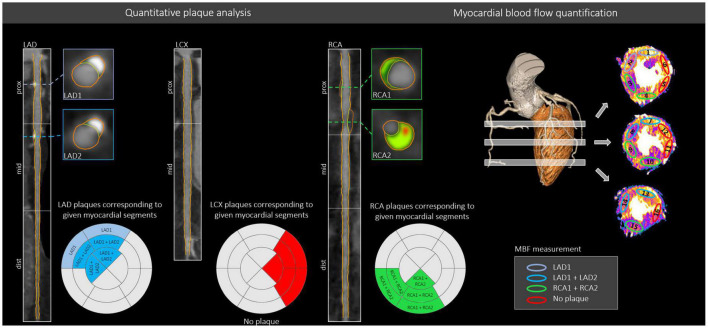
Comprehensive plaque assessment and the evaluation of myocardial ischemia based on CT images. A representative case of our study depicts the images of a 62-year-old male patient who underwent coronary CTA and DPCT imaging for the evaluation of CAD and corresponding myocardial ischemia. In this case, plaques were detected and quantified in the proximal LAD (LAD1), mid-LAD (LAD2) and proximal RCA (RCA1 + RCA2) coronary segments. All coronary plaques based on their location were matched for a given myocardial territory. MBF was quantified for all 16 myocardial segments using DPCT images. By creating a myocardial vessel territory map for each coronary segment, we could derive the total plaque volume that possibly influences the blood flow quantified as MBF on DPCT to any of the 16 analyzed LV territories (see Figure 2 for segmental classification). In this case, basal anterior (1) and basal anteroseptal (2) myocardial segments were influenced by the proximal LAD (LAD1) lesion, while the mid anterior (7), mid anteroseptal (8), apical anterior (13), and apical septal (14) myocardial segments were affected by two lesions: LAD1 and LAD2 (mid LAD segment) as they are located more distally. No plaques were present in the LCX. Regarding the RCA, two plaques were detected in the proximal RCA segment (RCA1 and RCA2), therefore all myocardial segments corresponding to the RCA were influenced by two plaques (RCA1 and RCA2). Plaque volumes of the lesions aligned for a given myocardial segment were summed and accounted for in the analysis, while the highest degree of lumen area stenosis of the corresponding lesions was included in the analysis. Light blue color indicates LV segments related to proximal LAD lesion, dark blue color indicates LV territories related to both proximal and mid-LAD lesions, whereas green color shows myocardial territories corresponding to the RCA lesions. LCX demonstrated no coronary lesions. ROI-s were placed in each myocardial segment on short-axial images. CAD, Coronary artery disease; CTA, CT angiography; DPCT, Dynamic perfusion CT; LAD, Left anterior descending; LCX, Left circumflex; LV, Left ventricle; MBF, Myocardial blood flow; RCA, Right coronary artery.

As highlighted above, there are conflicting results on whether luminal narrowing, plaque composition and vulnerability or plaque burden precipitate ischemia. This could originate from the high inter-vendor, inter-scanner, inter-protocol variability of coronary plaque assessment and from the methodology used for the characterization of ischemia. To our knowledge, there are currently no studies evaluating both myocardial ischemia and CAD quantitatively, on a segment level using CT imaging. Also, most of the former studies used visual assessment for detecting perfusion defects. However, quantitative methods are more reproducible and might provide a more detailed analysis of LV blood flow based on different perfusion markers. One of the largest challenges is the precise alignment of a myocardial territory to its feeding vessels. The most common approach is to calculate an accumulated PV for a given vessel, however this could not tailor unique variations in coronary anatomy and lesions on the distal coronary segments should not be taken into consideration when assessing perfusion in the basal myocardium. Authors of the CORE320 trial sought to assign coronary lesions to the corresponding myocardial segment taking anatomical variations and coronary dominance into account (18). For our segment-based approach, we defined all coronary artery segments that supply a given myocardial segment (16-segment model) based on dominance, segment location in relation to basal, mid-ventricular or apical regions (Figure 2). A representative case and the detailed description of our methodology are demonstrated on Figure 4. Using this approach, we aimed to overcome a main limitation of former studies which performed vessel-based analysis when evaluating ischemia. However, a distally localized lesion in the coronary vessel does not limit the flow of the most basal segments and this could substantially influence the results. Also, apical region of the heart can be supplied by several contributing vessel segments and therefore all of the lesions should be taken into consideration.

The discordance between stenosis severity and ischemia has been reported using both non-invasive and invasive methods. As previously described by Schuijf et al. in patients with obstructive CAD detected on coronary CTA, only 50% had ischemia using SPECT, while ischemia was detected in 15% of patients without obstructive CAD (4). Similarly, in the FAME study discrepancy was identified between anatomic and functional stenosis severity assessed by invasive angiography and FFR (22). Despite these findings, our current clinical management heavily relies on treating coronary lesions based on ischemic burden. However, total coronary plaque burden might step forward as the target of early interventions to stabilize HRPs, reduce the progression of coronary PV and luminal stenosis and thus ultimately to prevent adverse events.

Conflicting results regarding the relationship of plaques and ischemia may originate from the different capabilities of the modality utilized in the trials. CT has several advantages as compared with other techniques such as better spatial resolution, robust plaque assessment and reproducible quantitative measures of perfusion. CT imaging can define the hemodynamic significance of CAD by defining either lesion-specific ischemia using CT derived fractional flow reserve (FFR) or global ischemia on DPCT imaging. Radiation dose however still limits its use—especially using dynamic CT protocols—for a large subset of patients. Growing body of evidence suggests that anatomical information derived from CTA outperforms traditional ischemia testing for defining lesion-specific ischemia as obtained from invasive FFR. The CREDENCE trial demonstrated a strong association between atherosclerotic PVs, lumen size and invasive FFR (23). Our study provides unique insight in the interplay of coronary plaque burden, stenosis severity, HRP anatomy and corresponding myocardial ischemia on CTA. While HRP was linked to lesion-specific ischemia based on several trials (24), we did not see an association with reduced MBF on a segmental level. We found that considering all possible plaque on the feeding vessels of a given myocardial territory, total PV aggravates MBF or MBFi (per 10 mm^3^; β = −0.035, *p* < 0.01 for MBF and β = −0.0002, *p* < 0.01 for MBFi). This observation seems valid across different stages of stenosis severity and thus detailed plaque quantification could effectively guide secondary prevention therapy in a large spectrum of contemporary chest pain patients. Integrating plaque burden in the clinical CTA reports and thus in personalized patient management should be in the focus rather than luminal narrowing *per se*.

We acknowledge the limitations of our study. First, the sample size is limited after excluding patients with non-diagnostic image quality for quantitative plaque analysis or patients without intermediate stenosis. Excellent image quality is a prerequisite for quantitative plaque analysis. This could result in selection bias for our analysis. Quantitative plaque analysis is time-consuming and currently only a research tool, not used in routine clinical practice. However, experts of the field underline its role in risk prediction and tools are being developed for automated quantification in the near future. Also, our study is underpowered for the assessment of gender differences in CAD and corresponding ischemia or for outcome analysis.

## Conclusion

Total coronary PV was independently associated with myocardial ischemia based on MBF derived from DPCT imaging, while area stenosis and HRP were not. Incorporating these quantitative plaque characteristics in a comprehensive coronary CTA evaluation could improve the prediction of ischemic CAD, independently of lesion severity.

## Data availability statement

The data that support the findings of this study are available from the corresponding author, upon reasonable request.

## Ethics statement

The studies involving human participants were reviewed and approved by National Institute of Pharmacy and Nutrition—OGYÉI/719/2017. The patients/participants provided their written informed consent to participate in this study.

## Author contributions

BS, MK, PM-H, and BM contributed to conception and design of the study. BV, SB, and BS performed the measurements. BV, SB, MB, MV-N, FS, ÁJ, and BS contributed to patient enrollment. BV, SB, and MB organized the database. BS and MK performed the statistical analysis. BV and BS wrote the first draft of the manuscript. MK, MV-N, FS, SB, and MB wrote sections of the manuscript. All authors contributed to manuscript revision, read, and approved the submitted version.

## References

[1] Knuuti J Wijns W Saraste A Capodanno D Barbato E Funck-Brentano C 2019 ESC Guidelines for the diagnosis and management of chronic coronary syndromes. *Eur Heart J.* (2020) 41:407–77. 31504439 10.1093/eurheartj/ehz425

[2] Williams MC Kwiecinski J Doris M McElhinney P D’Souza MS Cadet S Low-Attenuation noncalcified plaque on coronary computed tomography angiography predicts myocardial infarction: results from the multicenter SCOT-HEART trial (Scottish Computed Tomography of the HEART). *Circulation.* (2020) 141:1452–62. 10.1161/CIRCULATIONAHA.120.049840 32174130 PMC7195857

[3] Xaplanteris P Fournier S Pijls NHJ Fearon WF Barbato E Tonino PAL Five-year outcomes with PCI guided by fractional flow reserve. *N Engl J Med.* (2018) 379:250–9. 10.1056/NEJMoa1803538 29785878

[4] Schuijf JD Wijns W Jukema JW Atsma DE de Roos A Lamb HJ Relationship between noninvasive coronary angiography with multi-slice computed tomography and myocardial perfusion imaging. *J Am Coll Cardiol.* (2006) 48:2508–14. 10.1016/j.jacc.2006.05.080 17174190

[5] Pontone G Rossi A Guglielmo M Dweck MR Gaemperli O Nieman K Clinical applications of cardiac computed tomography: a consensus paper of the european association of cardiovascular imaging-part I. *Eur Heart J Cardiovasc Imaging.* (2022) 23:299–314. 10.1093/ehjci/jeab293 35076061 PMC8863074

[6] Nieman K Balla S. Dynamic CT myocardial perfusion imaging. *J Cardiovasc Comput Tomogr.* (2020) 14:303–6. 10.1016/j.jcct.2019.09.003 31540820 PMC7064397

[7] van Rosendael AR Kroft LJ Broersen A Dijkstra J van den Hoogen IJ van Zwet EW Relation between quantitative coronary CTA and myocardial ischemia by adenosine stress myocardial CT perfusion. *J Nucl Cardiol.* (2017) 24:1253–62. 10.1007/s12350-016-0393-7 26860110 PMC5548828

[8] Eskerud I Gerdts E Larsen TH Simon J Maurovich-Horvat P Lonnebakken MT. Total coronary atherosclerotic plaque burden is associated with myocardial ischemia in non-obstructive coronary artery disease. *Int J Cardiol Heart Vasc.* (2021) 35:100831. 10.1016/j.ijcha.2021.100831 34258383 PMC8255815

[9] Diaz-Zamudio M Fuchs TA Slomka P Otaki Y Arsanjani R Gransar H Quantitative plaque features from coronary computed tomography angiography to identify regional ischemia by myocardial perfusion imaging. *Eur Heart J Cardiovasc Imaging.* (2017) 18:499–507. 10.1093/ehjci/jew274 28025263 PMC5837445

[10] Abbara S Blanke P Maroules CD Cheezum M Choi AD Han BK SCCT guidelines for the performance and acquisition of coronary computed tomographic angiography: a report of the society of cardiovascular computed tomography guidelines committee: endorsed by the north american society for cardiovascular imaging (NASCI). *J Cardiovasc Comput Tomogr.* (2016) 10:435–49. 10.1016/j.jcct.2016.10.002 27780758

[11] Iskandrian AE Bateman TM Belardinelli L Blackburn B Cerqueira MD Hendel RC Adenosine versus regadenoson comparative evaluation in myocardial perfusion imaging: results of the ADVANCE phase 3 multicenter international trial. *J Nucl Cardiol.* (2007) 14:645–58. 10.1016/j.nuclcard.2007.06.114 17826318

[12] Tanabe Y Kido T Uetani T Kurata A Kono T Ogimoto A Differentiation of myocardial ischemia and infarction assessed by dynamic computed tomography perfusion imaging and comparison with cardiac magnetic resonance and single-photon emission computed tomography. *Eur Radiol.* (2016) 26:3790–801. 10.1007/s00330-016-4238-1 26852220

[13] Achenbach S Moselewski F Ropers D Ferencik M Hoffmann U MacNeill B Detection of calcified and noncalcified coronary atherosclerotic plaque by contrast-enhanced, submillimeter multidetector spiral computed tomography: a segment-based comparison with intravascular ultrasound. *Circulation.* (2004) 109:14–7. 10.1161/01.CIR.0000111517.69230.0F 14691045

[14] Motoyama S Sarai M Harigaya H Anno H Inoue K Hara T Computed tomographic angiography characteristics of atherosclerotic plaques subsequently resulting in acute coronary syndrome. *J Am Coll Cardiol.* (2009) 54:49–57. 10.1016/j.jacc.2009.02.068 19555840

[15] Cerqueira MD Weissman NJ Dilsizian V Jacobs AK Kaul S Laskey WK Standardized myocardial segmentation and nomenclature for tomographic imaging of the heart. a statement for healthcare professionals from the cardiac imaging committee of the council on clinical cardiology of the american heart association. *Circulation.* (2002) 105:539–42. 10.1161/hc0402.102975 11815441

[16] Pontone G Baggiano A Andreini D Guaricci AI Guglielmo M Muscogiuri G Dynamic stress computed tomography perfusion with a whole-heart coverage scanner in addition to coronary computed tomography angiography and fractional flow reserve computed tomography derived. *JACC Cardiovasc Imaging.* (2019) 12:2460–71. 10.1016/j.jcmg.2019.02.015 31005531

[17] Nous FMA Geisler T Kruk MBP Alkadhi H Kitagawa K Vliegenthart R Dynamic myocardial perfusion CT for the detection of hemodynamically significant coronary artery disease. *JACC Cardiovasc Imaging.* (2022) 15:75–87. 10.1016/j.jcmg.2021.07.021 34538630 PMC8741746

[18] Cerci RJ Arbab-Zadeh A George RT Miller JM Vavere AL Mehra V Aligning coronary anatomy and myocardial perfusion territories: an algorithm for the CORE320 multicenter study. *Circ Cardiovasc Imaging.* (2012) 5:587–95. 10.1161/CIRCIMAGING.111.970608 22887690

[19] Liu T Yuan X Wang C Sun M Jin S Dai X. Quantification of plaque characteristics detected by dual source computed tomography angiography to predict myocardial ischemia as assessed by single photon emission computed tomography myocardial perfusion imaging. *Quant Imaging Med Surg.* (2019) 9:711–21. 10.21037/qims.2019.04.07 31143662 PMC6511719

[20] Driessen RS Stuijfzand WJ Raijmakers PG Danad I Min JK Leipsic JA Effect of plaque burden and morphology on myocardial blood flow and fractional flow reserve. *J Am Coll Cardiol.* (2018) 71:499–509. 10.1016/j.jacc.2017.11.054 29406855

[21] Min JK Chang HJ Andreini D Pontone G Guglielmo M Bax JJ. Coronary CTA plaque volume severity stages according to invasive coronary angiography and FFR. *J Cardiovasc Comput Tomogr.* (2022). 10.1016/j.jcct.2022.03.001 [Epub ahead of print]. 35379596

[22] Tonino PA Fearon WF De Bruyne B Oldroyd KG Leesar MA Ver Lee PN Angiographic versus functional severity of coronary artery stenoses in the FAME study fractional flow reserve versus angiography in multivessel evaluation. *J Am Coll Cardiol.* (2010) 55:2816–21. 10.1016/j.jacc.2009.11.096 20579537

[23] Stuijfzand WJ van Rosendael AR Lin FY Chang HJ van den Hoogen IJ Gianni U Stress myocardial perfusion imaging vs coronary computed tomographic angiography for diagnosis of invasive vessel-specific coronary physiology: predictive modeling results from the computed tomographic evaluation of atherosclerotic determinants of myocardial ischemia (CREDENCE) Trial. *JAMA Cardiol.* (2020) 5:1338–48. 10.1001/jamacardio.2020.3409 32822476 PMC7439215

[24] Danad I Raijmakers PG Driessen RS Leipsic J Raju R Naoum C Comparison of Coronary CT Angiography, SPECT, PET, and Hybrid Imaging for Diagnosis of Ischemic Heart Disease Determined by Fractional Flow Reserve. *JAMA Cardiol.* (2017) 2:1100–7. 10.1001/jamacardio.2017.2471 28813561 PMC5710451

